# Unforeseen uses of oral contraceptive pills: Exploratory study in Jordanian community pharmacies

**DOI:** 10.1371/journal.pone.0244373

**Published:** 2020-12-21

**Authors:** Muna Barakat, Raja’a Al-Qudah, Amal Akour, Najem Al-Qudah, Yahya H. Dallal Bashi

**Affiliations:** 1 Faculty of Pharmacy, Applied Science Private University, Amman, Jordan; 2 Faculty of Pharmacy, Department of Pharmacy, Al-Zaytoonah University of Jordan, Amman, Jordan; 3 School of Pharmacy, Department of Biopharmaceutics and Clinical Pharmacy, The University of Jordan, Amman, Jordan; 4 Faculty of Medicine, Al-Balqa Applied University, Al-Salt, Jordan; 5 School of Pharmacy, Queen’s University Belfast, Belfast, United Kingdom; Newcastle University, UNITED KINGDOM

## Abstract

**Background:**

The use of oral contraceptive pills (OCPs) as a birth control method is very common worldwide. OCPs have many other labeled non-contraceptive indications, and as a result there is an associated risk of improper use, as with any other medications. This study was designed to assess the unforeseen improper uses of OCPs observed by community pharmacists in Jordan.

**Method:**

A cross-sectional study design was conducted using a self-administered survey. A convenience sample (n = 380) of Jordanian community pharmacists, were recruited through social media resources. The survey included multiple-choice and open-ended questions. Descriptive statistics and correlation analyses were completed using SPSS.

**Results:**

More than half of the recruited pharmacists (55.3%) were female, and the mean age of the participants was 32.58 ± 9.94. The majority of the pharmacists (85%) had good knowledge about the non-contraceptive indications of OCPs. About 53% of them confirmed their exposure to cases of the improper use of OCPs. About 67.5% of the pharmacists who confirmed exposure to such cases, reported the topical use of OCPs for the enhancement of hair growth. Around 15% of those pharmacists stated that OCPs were used to give negative results for addictive drug screening tests. In the event that the pharmacists suspected improper use, more than 90% suggested they would refrain from dispensing the pills.

**Conclusion:**

This study has spotlighted many unforeseen uses of OCPs in Jordan and highlighted the need for restricted national regulations on the monitoring of OCP prescription/selling patterns in Jordan by policymakers. Moreover, there is a need for the establishment of national educational programs for the Jordanian community regarding the safe proper use of OCPs.

## Introduction

Since the 1960s, oral contraceptive pills (OCPs) have become globally abundant and usable by the vast majority of women [1, 2]. According to the data, nearly all women prefer to use OCPs [3] and it is considered one of the most frequently used contraception methods [3, 4]. In addition, OCPs have many other indications apart from their purpose as a birth control method use, such as regulation of menstruation, dysmenorrhea, endometriosis, acne treatment, and reducing the risk of endometrial, ovarian, and colon cancer [5]. In five European countries, a comprehensive study found that OCPs were rated as the most widely used method of contraception among women [1]. In Jordan, OCPs are one of the most common birth control methods, which is used by 60% of Jordanian women [6].

Improper use of medications could include misuse and abuse practices. According to the National Institute on Drug Abuse, misuse of prescription drugs means “taking a medication in a manner or dose other than prescribed; taking someone else’s prescription, even if for a legitimate medical complaint such as pain; or taking a medication to feel euphoria” [7]. Drug abuse is the use of a substance for nontherapeutic purposes to experience psychotropic (e.g., euphoric, sedative, or anxiolytic) effects [8]. OCPs are highly susceptible to be used improperly as these pills are readily available in the market and can be sold without a prescription in Jordanian Pharmacies [9]. OCPs might be improperly used, or used for other unintended indications, or used for a longer duration than intended without medical advice [9]. Besides, many types of OCPs suit a variety of individuals depending on their intended need and use. For example, some women use OCPs on a long term basis to delay aging and enhance femininity, since this is recommended by many beauty centers (unauthorized health care providers) [9].

Furthermore, the appropriateness of using OCPs depends on several factors, including a woman’s health status, age, medical history, smoking status, and other related factors [10]. Jordan pharmacy law has no regulations to restrict the prescription of OCPs, therefore, these pills can be obtained without a prescription from community pharmacies [6]. A study by Bardaweel et al. found that more than 75% of women in Jordan had consulted a doctor and received a prescription before using OCPs.

A study conducted in the United Arab Emirates found that more than 92% of community pharmacists did not ask the patient any question to assess their eligibility to use oral contraceptives [11]. Whereas, both the United States and the United Kingdom have well-designed protocols to guide pharmacists in proper and safe OCPs handling [12, 13]. It has been reported that community pharmacists play a crucial role in the management of hormonal contraceptive usage and in avoiding any improper use of the products among women [12, 13].

On the other hand, studies from developing countries concerning pharmacists’ involvement in dispensing OCPs have shown that there are minimal assessments and suboptimal screening for the safe use of these products by patients [14–16]. Hence, to the authors’ knowledge, there is no documentation of OCP misuse and abuse cases and nothing regarding how pharmacists can manage it. Thus, this study aims to assess the unforeseen improper uses of OCPs observed by community pharmacists in Jordan.

## Materials and methods

### Study design and participants

This study followed a descriptive cross-sectional design and the objectives were addressed via an online survey. The study was conducted in Jordan from 8th June to 27th July 2020. The online survey was developed and validated by clinical researchers to solicit anonymous responses, which were treated confidentially. The eligible participants, which included Jordanian pharmacists and trainees (who were working/training in community pharmacy) were recruited using a convenience sampling method. The inclusion criteria were explained at the start of the survey, which stated: “If you are working or training at a community pharmacy, please let us know if you would like to participate in this survey”.

Participants were recruited through social media platforms (Facebook, WhatsApp, LinkedIn, and Twitter). Participants were advised that their participation in the study was voluntary and did not pose any risks. A written participant consent statement “Your participation in completing this questionnaire is highly appreciated” was given to the participants at the beginning of the survey. If the participants were willing to proceed with the survey, they approved their consent. If not, they selected “disagree to participate” and did not continue with the survey questions. Potential participants who completed the survey were considered to have given informed consent for their participation in the study. The Ethical approval for the study was obtained from the Faculty of Pharmacy, Applied Science Private University (Approval number: 2019-PHA-14).

### Survey development, validation, and reliability

The online survey was developed after reviewing related validated surveys in the literature [6, 17, 18], and was designed using the general principles of good survey design [18]. Several sources were used to generate a pool of questions considered to be relevant to the objectives of the study [18]. The online survey was finally prepared using Google Forms, and although it was constructed in English, it was delivered to the participants in Arabic, the formal language of Jordan. The survey contained multiple-choice and open-ended questions and was designed to be completed within 7–10 minutes.

To ensure face validity, the first draft of the survey was evaluated by fifteen independent academic staff members who had previous experience in OCPs related work and research studies. A statistician was also involved at this stage of the evaluation. All the provided comments and feedback were considered and incorporated where appropriate to prepare the final version of the survey. The survey was then translated from English into Arabic and then back-translated by two senior academic staff members who were considered fluent in both languages. The questions were free from medical jargon or difficult terminology. Finally, the survey was piloted via a sample of 25 academic and 25 non-academic participants. This stage of the study was conducted to enhance clarity, readability, understandability, and confirm the study’s applicability to Jordanian community pharmacists. Internal consistency reliability was tested by the Cronbach’s alpha coefficient, which equaled 0.89.

The final version of the survey contained three parts. Part A comprised of twelve questions, which included sociodemographic information. Part B consisted of four questions comprising details of the participants’ knowledge and beliefs toward the use of OCPs. The questions regarding knowledge were mainly concerned with the presence of non-contraceptive indications and the side effects of OCPs. Here, the participants were able to choose more than one option. Part C focused on the pharmacist's experience and practice towards the improper use of OCPs. This consisted of ten multiple-choice questions and one open short essay. The pharmacists who confirmed their exposure to suspected cases of the improper use of OCPs provided more detailed answers about their observations in the open-ended question, which presented us with general themes. In the last section, the participants were asked if they had been exposed before to any improper use practices relating to OCPs. When they answered “yes”, an open-ended question was designed to allow the participant to explain in detail their experience of the improper use of OCPs. In addition, they were also asked about the proper methods to deal with such practices. A copy of the final version of the survey (in both the original language and English) can be seen in S1 and S2 Appendixs.

### Sample size

The most recent statistics released by the Jordanian Pharmacist’s Association (JPA) show that there were 22,667 registered pharmacists by February 2019 [19]. This statistical report reveals the Jordanian pharmacists' demographics, according to gender (i.e. females n = 14,587, 64%), the province where they work (i.e. Amman n = 15,866, 70%), mean age (30.7 ± 8.2 years old) and also showed that the majority had a bachelor’s degree (n = 17,667, 77.6%) [19]. Based on this data, the sample size was calculated using a margin of error of 5%, a confidence level of 95%, and a response distribution of 50%, giving a minimum sample size of 378 pharmacists [20]. The decision was made to increase the number to around 380 pharmacists to take into account missing responses and other unknown issues that might arise.

The sample was taken from the total number of registered pharmacists in Jordan (a homogeneous sample). According to the sample size calculations, the convenience homogeneous sample represents the whole population and it can be generalized on a national level [21].

### Statistical analyses

The completed surveys were extracted from Google Forms as an Excel sheet and were then exported to Statistical Package for Social Sciences version 24.0 (SPSS® Inc., Chicago, IL, USA) for the statistical analysis. The descriptive statistics included percentages, means, and frequency distribution, which were calculated for each question. Descriptive and univariate correlation analyses using Pearson correlation coefficient (r) were used for the correlation, which was conducted at a 5% significance level. A *p*-value of < 0.05 represented a significant difference. The answers to the open-ended question were transferred to an Excel sheet, analyzed and categorized for themes, and presented as frequencies and percentages. Factors affecting the improper use of OCPS were analyzed using simple and multivariate linear regression.

## Results

### Sociodemographic characteristics

Out of the total 383 completed questionnaires, three forms (0.8%) were excluded from the study due to incomplete responses. Accordingly, 380 (99.2%) of the answered questionnaires were included in the study analysis. More than half of the participating pharmacists were female (n = 210, 55.3%). The mean age of the participants was (32.58 ± 9.94) and the majority held a bachelor’s degree (n = 274, 72.2%).

Regarding the participants’ experience in community pharmacy, half of them (n = 192, 50.5%) had been working for a relatively short period (i.e. less than 5 years). Most of the participants were working in pharmacies located in the capital of Jordan Amman" (n = 250, 65.8%). Regarding the participants' perceptions, around 84% (n = 319) of the pharmacy customers were classified as a middle-income social class, regardless of the province where they worked.

In terms of the available facilities around the pharmacy, less than half (n = 157, 41%) of the participants reported that there were sports gyms, while 60.8% (n = 231) mentioned that there were beauty centers around the pharmacy (Table 1).

**Table 1:** Sociodemographic characteristics of the participants (n = 380).

Characteristic	n	%
**Age (mean ±SD)**	32.58± 9.94	
**Gender**		
• Female	210	55.3
• Male	170	44.7
**Education**		
• Bachelor’s degree	304	80.0
• Pharmacy student +trained in a pharmacy	35	9.2
• Postgraduate degree	29	7.6
• Diploma	12	3.2
**Social status**		
• Single	193	50.8
• Married	175	46.1
• Divorced/ Widowed	12	3.1
**Number of children**		
• No children yet	26	6.8
• 1 to 3	124	32.6
• 4 to 6	37	9.7
• Not applicable (single)	193	50.8
**Years of experience**		
• <5	192	50.5
• 5 to 10	64	16.8
• 11 to 15	58	15.3
• 16–20	24	6.3
• >20	42	11.1
**Position in the pharmacy**		
• Employee pharmacist	152	40
• Trainee in a pharmacy	82	21.6
• Pharmacy owner and employee at the same time	61	16.1
• Pharmacy owner	22	5.8
• Others	50	13.2
**Province where you work**		
• The capital of Jordan (Amman)	250	65.8
• Irbid	43	11.3
• Zarqa	46	12.1
• Others	41	10.7
**The most common social class distribution of the pharmacy customers (more than one option was allowed)**		
• Low	122	32.1
• Middle	319	83.9
• High	41	10.8
**The presence of a sports gym around the pharmacy**		
• Yes	157	41.3
• No	191	50.3
• Not sure	32	8.4
**The presence of the beauty center near your pharmacy**		
• Yes	231	60.8
• No	121	31.8
• Not sure	28	7.4

### Knowledge and beliefs of pharmacists about the non-contraceptive uses of OCPs

**Fig 1:**
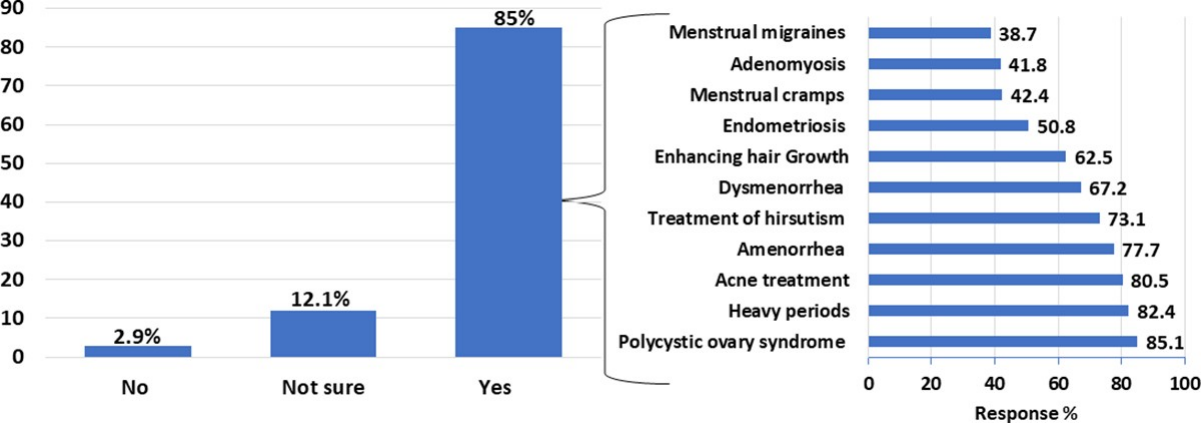
Pharmacists’ knowledge about the presence of non-contraceptive indications for OCPs (n = 380). Among the participants who agreed with the presence of non-contraceptive indications for OCPs (n = 323, 85%), the horizontal column chart demonstrates the chosen indications.

The majority of the participating pharmacists (n = 323, 85%) agreed that OCPs have non-contraceptive indications (Fig 1). More than 80% of those pharmacists stated that OCPs could be used for the treatment of polycystic ovary syndrome, heavy periods and acne (Fig 1). Assessing the pharmacist’s knowledge of the possible side effects of OCPs revealed that OCPs are associated with mood changes, increased body weight, breast enlargement and venous thromboembolism in 95.3%, 92.1%, 78.7% and 77.6%, respectively. Only, 28.7% of the participants knew that OCPs could cause gall bladder disease (S1 Fig).

### Unforeseen uses of OCPs

**Fig 2:**
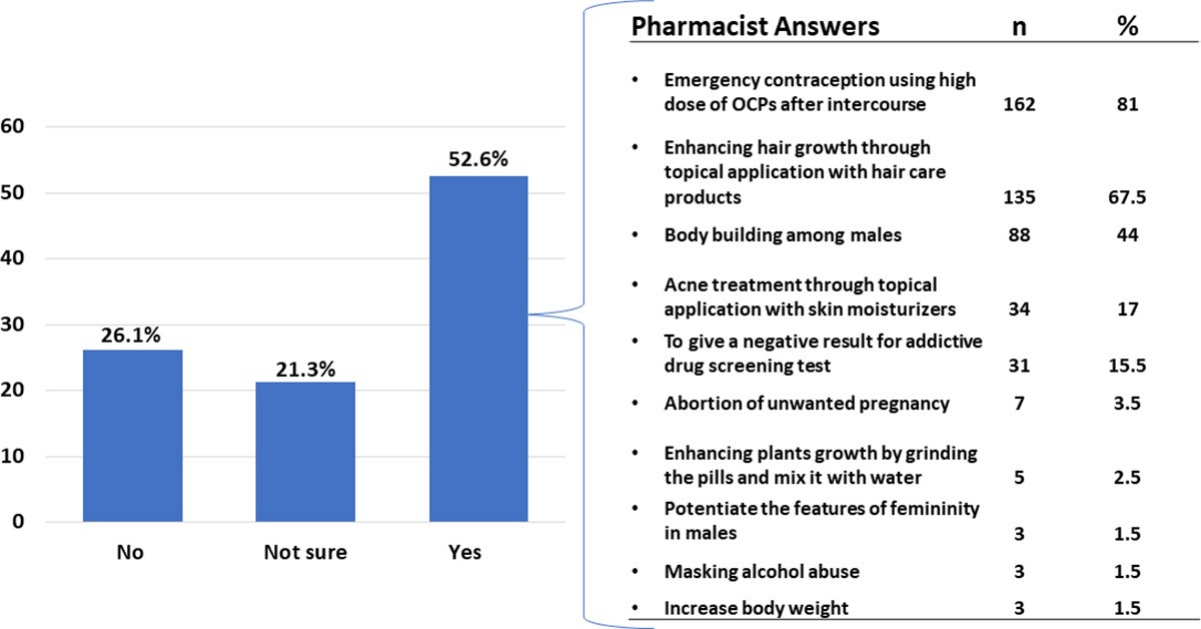
The responses of pharmacists after asking them if they had ever been exposed to OCPs’ improper use cases (n = 380). Among the participants who answered “YES” (n = 200, 52.6%) with the presence of OCPs improper use, the participants were able to write detailed answers using an open-ended question and presented as main themes in the table.

The participating pharmacists were asked, “if they had ever been exposed to OCPs improper use cases”. Half of them confirmed their exposure to such cases (n = 200, 53%) (Fig 2), and those pharmacists provided more detailed answers about their observations using an open-ended question, which presented general themes. The majority of those pharmacists (n = 162, 81%) reported that many kinds of OCPs were used by females as emergency contraception (i.e. unlicensed OCPs), who used a very high dose of these OCPs directly after intercourse. A further ten participants suggested that OCPs were being taken 15 pills at a time, or one full pack in one go, the following intercourse. Other participants stated that OCPs were used topically for hair growth enhancement and acne treatment. Around 15% of the pharmacists reported that OCPs were being used to give a negative result for addictive drug screening tests. More detailed answers are summarized in Fig 2.

The pharmacists who confirmed their exposure to the improper use of OCPS also revealed that both genders are susceptible. More than 60% of those pharmacists reported that those who improperly used OCPs were a cohort of strangers and regular customers (n = 139, 69.5%) and were aged 20–30 (n = 151, 75.5%). Friends were rated the most common source (n = 182, 91%) that encourage them to use OCPs. The OCP “levonorgestrel with ethinyl estradiol” was documented as the most susceptible product for improper use (n = 157, 78.5%), according to the participating pharmacists’ experience, followed by “drospirenone with ethinyl estradiol” and “norethisterone alone”, at 64% and 62%, respectively (Table 2).

**Table 2:** Reported information about the OCPs improper users and the most commonly used products, according to the pharmacist’s observations for the OCPs improper use cases (n = 200).

Question	n	%
**Which gender group is/are more vulnerable to OCPs improper use?**		
• Males	45	22.5
• Females	61	30.5
• Both males and females	94	47
**Which age group is/are more vulnerable to OCPs improper use?**		
• <20	9	4.5
• 20–30	151	75.5
• 31–40	35	17.5
• 41–50	2	1
• >50	3	1.5
**Which group is/are more vulnerable to OCPs improper use?**		
• Strangers	55	27.5
• Regular (known) pharmacy visitors	6	3
• A mix of both strangers and visitors	139	69.5
**What is the source of information that guides the OCPs improper user to use it? (more than one option was allowed)**		
• Friend	182	91
• Social media	159	79.5
• Sport gym	147	73.5
• Beauty center	145	72.5
• Family	117	58.5
• Television	46	23
• Pharmacist	39	19.5
• Physician	39	19.5
**According to your experience, which of the following OCPs products are more vulnerable to improper use? (more than one option was allowed)**		
• levonorgestrel + ethinyl estradiol	157	78.5
• drospirenone+ ethinyl estradiol	128	64
• norethisterone alone	124	62
• cyproterone+ ethinyl estradiol	102	51
• desogestrel+ ethinyl estradiol	48	24
• norethisterone +estriol + ethinyl estradiol	48	24
• norgestrel+ ethinyl estradiol	40	20
• dydrogesterone+ ethinyl estradiol	37	18.5
• norethindrone+ ethinyl estradiol	23	11.5

### Pharmacist attitude and behavior towards OCPs the improper use of OCPs

Eighty-three percent (n = 317) of the participating pharmacists disclosed that they had dispensed OCPs without prescription. More than 40% (n = 154) of the study’s participants stated that those who improperly used OCPs directly asked and admitted their need for these pills, while 60% (n = 127) of pharmacists were able to suspect that an OCP customer could be an improper user from their facial and body language. In the case of suspicion for improper use, most of the pharmacists (n = 355, 93.4%) affirmed that they would not dispense these medications. Therefore, they would rather advise/clarify the side effects of these pills in order to limit the improper use of OCPs (n = 364, 95%). Further solutions that have been suggested by the participant pharmacists to limit the improper uses of OCPs are detailed in Table 3.

**Table 3:** Pharmacist attitude and behavior towards OCPs improper use (n = 380).

Question	n	%
**In case of confirmation of OCPs improper use from certain pharmacy visitors, would dispense it for them?**		
• No	355	93.4
• Yes	25	6.6
**How do you usually recognize the improper user of OCPs? (more than one option was allowed)**		
• They ask directly and admit their needs	154	40.5
• I can recognize them from the facial impressions and body language	127	33.4
• They come regularly to the pharmacy (known people) asking for the same products.	101	26.6
• I cannot recognize them	41	10.8
**If OCPs were not prescribed, what are the methods used by pharmacists to limit OCPs improper use? (more than one option was allowed)**		
• Advise and clarify the side effects of these pills	364	95.8
• Requesting a prescription	304	80.0
• Refusal to dispense or claim that the product is not available	290	76.3
• Referring patient to the physician	250	65.8
• Conduct awareness campaigns and pamphlets to raise awareness	215	56.6
• Working with JPA to solve the problem	199	52.4
• Hiding product from the shelf	198	52.1
• Report the improper use cases for the pharmacovigilance department of JFDA	151	39.7
• Do nothing	27	7.1
• Calling police	24	6.3

Multivariate logistic regression outcomes (Table 4) showed a positive significant correlation *(p*< 0.05) between the pharmacist’s exposure to the improper use of OCPs and the following: the extent of the pharmacist’s experience (in years), the relatively low social class distribution of the pharmacy’s customers, and the presence of sport and beauty centers around the pharmacy.

**Table 4:** Summary of the linear regression analysis (single and multivariant) to assess factors associated with the exposure of participating pharmacists to OCPs improper use cases.

Independent factors	Single Linear regression			Multivariable regression		
	Beta	*p*-value		Beta	*p*-value	
**Age**	-0.148	**0.004**		-0.148		0.216
**Gender**						
• Female	Reference					
• Male	-0.143	**0.005**		-0.068		0.245
**Years of experience**						
• <5years	Reference					
• >5years	0.779	**0.001**		0.705		**<0.001**
**Province of the work**						
• The capital (Amman)	Reference					
• Outside the capital	-0.073	**0.05**		-0.051		0.131
**Social class distribution of the pharmacy customers**						
• Low	Reference					
• Moderate/high	-0.160	**0.002**		-0.163		**0.002**
**Presence of Sports Gym around the pharmacy**						
• Yes	Reference					
• No	-0.152	**0.005**		-0.162		**0.033**
**Presence of beauty center around the pharmacy**						
• Yes	Reference					
• No	-0.182	**0.008**		-0.192		**0.041**

Significance (*p*<0.05) presented in bold numbers.

## Discussion

With the rapid increase in the use and availability of OCPs, their efficacy and safety have become of paramount importance among users around the world, including Jordanians [6]. In this study, most of the improper uses of OCPs were unforeseen and were subsequently encountered by the participating community pharmacists. This highlights the pharmacist’s knowledge and practices towards OCPs and their role in the management of the improper use of medications.

The study findings have revealed that pharmacists and pharmacy trainees showed good knowledge about OCPs, who were aware of the non-contraceptive uses and the most common side effects of OCPs. Similar to these results, an Iranian study showed that more than 90% of pharmacists have identified the non-contraception use of OCPs and their side effects [22]. Also, a study in the United Arab Emirates showed that the majority of pharmacists could identify the most common side effects of OCPs, such as weight gain and mood fluctuations [23]. Moreover, many studies have focused on the pharmacists' role in counseling on the effective and safe use of OCPs, screening for contradictions, and drug-drug interactions [11, 12, 24–26]. Our findings demonstrate that the majority of the pharmacists refused to dispense OCPs in suspicious improper use cases and that they would rather provide counseling about the side effects of improper use, ask for a physician’s prescription or encourage physician’s referral, and/or report the incident to the Jordan Pharmacist Association (JPA) and the Jordan Food and Drug Administration (JFDA). However, the vast majority of community pharmacists in this study dispensed OCPs without prescription. Such practice is against the national regulations by the JPA or JFDA [27] and could directly contribute to the improper use of medications such as OCPs and antibiotics, etc. According to the literature, many contributing factors could explain these practices, which include financial considerations, since pharmacists need to sell the medications, or, they may be promoted by pharmaceutical companies [28]. This issue is very important at the national level, hence, many studies have investigated the possibility of changing the status of OCP to be an over the counter medication and expanding the scope of pharmacists’ practice beyond counseling and education to prescribing [29–32]. This suggests that there is a need for strict regulations and guidance to control any possibility of improper medication use.

Remarkably, most of the documented cases of the improper use of OCPs in this study have been rarely recognized in previous studies. In an Iranian study, OCP misuse was found to increase the risk of cerebral and venous thrombosis [33]. The most common causes of misuse (35%) were to delay menstruation to be able to perform religious customs, such as fasting and pilgrimage, to conduct family planning without prescription (30%), or the management of dysfunctional uterine bleeding without prescription (14%). In Sri Lanka, OCP overdose was used as a method of intentional self-poisoning in a women of young age, especially in the first year of marriage [34]. Other older studies have shown misuse related to missing the pill and increasing the risk of unintended pregnancy [35, 36]. The most reported case of improper OCP use by our study’s pharmacists was using a very high dose of OCPs at once after intercourse as emergency contraception. This could be due to the refusal of the JFDA to agree on the registration of emergency contraception (also known as plan B), due to recommendations from the House of Fatwa (Religious institution) [37]. On the other hand, the WHO mentioned in their last updated ‘Family Planning Handbook’ the allowed pill formulations and dosing for emergency contraception [2]. This guidance did not allow the use of a very high dose (i.e. one pack of OCPs), except for 0.03 and 0.0375 mg of levonorgestrel alone and 0.075 mg norgestrel alone, which are not available in Jordan [2].

Moreover, we found that OCPs were abused in Jordan to mask the results of addiction drug urine tests. It was reported that several adulterants could be added to urine in order to give a false-negative drug test, which could hinder efforts to monitor illicit drug use [38], which include oxidizing chemicals, such as nitrite or peroxide, as well as non-oxidizing chemicals. Hajhashemi et al. (2007) conducted an in vitro and in vivo study assessing the interaction of OCPs (ethinylestradiol, levonorgestrel (LN), and both of them) at a high dose with a urine morphine diagnostic test, after reporting plenty of claims about this issue [39]. The results of that study confirmed the absence of such an interaction, which strongly suggests there is a need to stop misusing these medications. However, such practices are still available according to our study findings, which should highlight the need for further future studies to better understand this issue and devise suitable recommendations for policymakers.

A few participants reported the use of OCPs for the enhancement of female features. Studies showed that the use of OCPs, in this case, may indicate a transgender tendency in males [40, 41]. This low incidence is expected since homosexuality is still socially unacceptable and the lesbian, gay, bisexual, and transgender (LGBT) community in Jordan face a great deal of discrimination, and stigmatization, in spite of the fact that the country’s laws ended the criminalization of homosexuality since 1951 [42]. Improper users of OCPs tend to be young customers (of both genders) and the most commonly misused pills are the most prescribed OCPs in Jordan, due to their accessibility and affordability [43].

The reporting of the improper usage of OCPs was correlated to many factors according to our study results, including the extent of the pharmacist’s experience. Pharmacist experience may empower their professionalism and their capability to differentiate the cases of proper/improper use of medication [44]. Another affecting factor was the low social class distribution of pharmacy customers. Most published studies have measured the effect of socio-economic distribution on substance use (e.g. illicit drugs and alcohol), whereas there has been nothing on medications such as OCPs, which reveals that “low social status report more environmental challenges and less psychosocial resources and that this can lead to feelings of hopelessness and a loss of coping ability” [45]. This could explain the improper use of OCPs to mask the test of illicit drug use.

Finally, In Jordan, the JFDA confirmed that OCPs should be dispensed to the patient under prescription. To this end, it has published a list of non-prescription medications [46]. However, a lack of monitoring makes OCPs accessible through community pharmacists without a prescription. This uncontrolled access and lack of monitoring of their consumption might make OCPs more susceptible to improper use, which could ultimately lead to an increase of side effects and complications [47]. However, there is scarce documentation on cases of the improper use of OCPs.

### Strengths and limitations

This has been the first large study in Jordan to assess various patterns of the improper uses of OCPs by both Jordanian females and males, which are encountered in the community pharmacy setting. The first limitation of this study was the participant self-selection process. The survey was conducted online due to the novel coronavirus pandemic that started around January of 2020 [48], along with the public quarantine (COVID-19 related) currently imposed in Jordan. Hence, only people who use the Internet and other social media platforms were able to participate. Another limitation is the use of convenient non-random sampling. However, our sample was fairly representative of the pharmacists in Jordan where most of the practitioners are females, residing in Amman.

## Conclusion

This study spotlighted many unforeseen improper uses of OCPs in Jordan. Findings from this study emphasize the important role that community pharmacists have in identifying and preventing the improper uses of OCPs and also highlights the need for applying more strict monitoring procedures on the OCP handling process by both policymakers and drug regulatory institutions in Jordan. Also, the establishment of national educational programs for the Jordanian community about the safe and proper use of OCPs should be implemented. To this end, we recommend performing studies that deeply explore the reasons behind the improper use of OCPs by the users themselves, with a view to evaluate the effect of awareness campaigns about improper use, and their effects on improper use rates and prescription patterns, in collaboration with JPA and JFDA.

## Supporting information

S1 Appendix Unforeseen uses of oral contraceptive pills: Exploratory study in Jordanian community pharmacies survey (English). (DOCX)

S2 Appendix Unforeseen uses of oral contraceptive pills: Exploratory study in Jordanian community pharmacies survey (Arabic). (DOCX)

S1 Fig The percentage of pharmacist knowledge about the OCPs side effects (n = 380). (TIF)
