# Supplementary material for: Unforeseen uses of oral contraceptive pills: Exploratory study in Jordanian community pharmacies
Source: PLoS One. 2020 Dec 21;15(12):e0244373. doi: 10.1371/journal.pone.0244373 (PMC7751968; doi:10.1371/journal.pone.0244373)
Supplement: S1 Appendix — (DOCX) [file pone.0244373.s001.docx]

**Unforeseen uses of oral contraceptive pills: Exploratory study in Jordanian community pharmacies**

We are a group of Jordanian researchers and we would like to invite you to fill this questionnaire, which aims assess the patterns of unforeseen improper uses of oral contraceptive pills (OCPs) observed by the community pharmacists in Jordan. It will be an observational study in collaboration with the community pharmacists.

The questionnaire consists of three sections only, which will require 7-10 minutes of your time to answer them, knowing that the questionnaire does not require writing the name or any other private information. The information will be treated in strict confidence and will be used for the purposes of scientific research.

Note:

According to the National Institute of Drug Abuse, misuse of prescription drugs means “taking a medication in a manner or dose other than prescribed; taking someone else’s prescription, even if for a legitimate medical complaint such as pain; or taking a medication to feel euphoria” (NIDA, 2020)

Drug abuse is defined as the use of a substance for a purpose not consistent with legal or medical guidelines (WHO, 2006)

**If you are working or training at a community pharmacy, please let us know if you would like to participate in this survey. Your participation in completing this questionnaire is highly appreciated**

Agree to participate

Disagree to participate

**Part 1. Demographic data for the participants**

- **Gender**
- Female
- Male

- **Age ………………………………years**
- **The highest education levels**
- Diploma
- Bachelor's degree
- Postgraduates degree
- Pharmacy student +trainer in a pharmacy
- **Marital status**
- Single
- Married
- Divorced/Widowed
- **Number of children**
- No children yet
- 1-3
- 4-6
- >6
- Not applicable (single)
- **Years of experience**
- <5
- 5-10
- 11-15
- 16-20
- >20
- **Which of the following best describes your position in the community pharmacy?**
- Pharmacy owner
- Employee pharmacist
- Pharmacy owner and employee at the same time
- Trainee in a pharmacy
- Other
- **Province where you work**
- The capital of Jordan (Amman)
- Irbid
- Zarqa
- Al-Salt
- Al-Mafraq
- Alkarak
- Madaba
- Jarash
- Ajloun
- Aqaba
- Mea'an
- Tafilaa
- **In general, the most common social class distribution of the pharmacy customers is (You can choose more than one option):**
- Low income
- Middle income
- High income
- **Is there any sports gym around your pharmacy?**
- Yes
- No
- I am not sure
- **Is there any beauty center near your pharmacy?**
- Yes
- No
- I am not sure

**Part 2. Knowledge and beliefs of pharmacist about OCPs uses**

- **According to your knowledge and opinion, Does OCP have any non-contraceptive indications?**

Yes

No

I am not sure

**If you answered yes in the previous question, please proceed with the next question**

- **According to your knowledge, what are the possible non-contraceptive uses of OCPs?**

|  | True | False |
| --- | --- | --- |
| Acne treatment |  |  |
| Enhancing Hair Growth |  |  |
| Heavy periods |  |  |
| Menstrual migraines |  |  |
| Menstrual cramps |  |  |
| Amenorrhea |  |  |
| Endometriosis |  |  |
| Adenomyosis |  |  |
| Treatment of hirsutism |  |  |
| Dysmenorrhea (painful menstruation) |  |  |
| Poly Cystic Ovary Syndrome |  |  |

- **What are the possible side effects of OCPs?**

|  | True | False |
| --- | --- | --- |
| Reduce Libido |  |  |
| Night Sweats |  |  |
| Increased Weight |  |  |
| Mood Changes |  |  |
| Breast Enlargement |  |  |
| Unintended hair growth |  |  |
| Venous thromboembolism |  |  |
| Breast cancer |  |  |
| Cervical cancer |  |  |
| Gallbladder disease |  |  |

**Part 3. A. Pharmacist experience and practice towards OCPs improper use**

- **According to experience, which is more common?**
- Dispensing OCPs with prescription
- Dispensing OCPs without prescription (as OTC)
- **Had you ever been exposed to any improper use cases of OCPs?**
- Yes
- No
- I am not sure

**If you answered “Yes” in the previous question, please answer the following questions**

- **What were those improper uses among the users, please mention them ………………………………………………………………………………………………………………………………………………………………………………………………………………………………………………………………………………………**
- **Which gender group is/are more vulnerable to improper use?**
- Males
- Females
- Both males and females
- **Which age group is/are more vulnerable to improper use?**
- <20
- 20-30
- 31-40
- 41-50
- >50
- **Which group is/are more vulnerable to improper use?**
- Strangers
- Regular (known) pharmacy visitors
- A mix of both strangers and visitors
- **What is the source of information that guides the OCPs improper user to use it? (you can choose more than one option)**
- Friend
- Social media
- Family
- Beauty center
- Sport gym
- Television
- Pharmacist
- Physician
- **According to your experience, which of the following OCPs products are more vulnerable to improper use? (you can choose more than one option)**
- Levonorgestrel + Ethinyl Estradiol (e.g. Microgynon)
- Desogestrel+ Ethinyl Estradiol (e.g. Marvelon)
- Norethindrone+ Ethinyl Estradiol (e.g. Kliogest)
- Dydrogesterone+ Ethinyl Estradiol (e.g. Femoston)
- Drospirenone+ Ethinyl Estradiol (e.g. Yasmin)
- Cyproterone+ Ethinyl Estradiol (e.g. Diane)
- Norgestrel+ Ethinyl Estradiol (e,g, Ovral)
- Norethisterone +Estriol + Ethinyl Estradiol (e.g. Trisequens)
- Norethisterone ALONE (e.g. Primolut Nor)

**3.B. Pharmacist attitude towards OCPs improper use**

- **In case of confirmation of OCPs improper use from certain pharmacy visitors, would dispense it for them?**
- Yes
- No
- **How do you usually recognize the improper use of OCPs? (you can choose more than one option)**
- They come regularly to the pharmacy (known people) asking for the same products.
- I can recognize them from the facial impressions and body language
- They ask directly and admit their needs
- I cannot recognize them
- **If OCPs were not prescribed, what are the methods used by pharmacists to limit OCPs' misuse and abuse? (You can choose more than one option)**
- Refusal to dispense or claim that the product is not available
- Advise and clarify the side effects of these pills
- Requesting a prescription
- Hiding product from the shelf
- Referring patient to the physician
- Working with JPA to solve the problem
- Report the improper use cases for the pharmacovigilance department of JFDA
- Conduct awareness campaigns and pamphlets to raise awareness
- Calling police
- Do nothing
