# Supplementary material for: Unforeseen uses of oral contraceptive pills: Exploratory study in Jordanian community pharmacies
Source: PLoS One. 2020 Dec 21;15(12):e0244373. doi: 10.1371/journal.pone.0244373 (PMC7751968; doi:10.1371/journal.pone.0244373)
Supplement: S2 Appendix — (DOCX) [file pone.0244373.s002.docx]

**الاستخدامات غير المتوقعة لأقراص منع الحمل: دراسة استكشافية في صيدليات المجتمع الأردني**

نحن مجموعة من الباحثين الأردنيين ونود أن ندعوكم لملء هذا الاستبيان ، الذي يهدف إلى تقييم أنماط الاستخدامات غير المتوقعة لحبوب منع الحمل التي لاحظها صيادلة المجتمع في الأردن. ستكون دراسة قائمة على الملاحظة بالتعاون مع صيادلة المجتمع.

يتكون الاستبيان من ثلاثة أقسام فقط ، والتي تتطلب من 7 إلى 10 دقائق من وقتك للإجابة عليه ، مع العلم أن الاستبيان لا يتطلب كتابة الاسم أو أي معلومات خاصة أخرى. سيتم التعامل مع المعلومات بسرية تامة وسيتم استخدامها لأغراض البحث العلمي.

ملحوظة:

وفقًا للمعهد الوطني لتعاطي المخدرات ، فإن إساءة استخدام العقاقير الموصوفة تعني "تناول دواء بطريقة أو جرعة غير موصوفة ؛ أو أخذ وصفة طبية من شخص آخر حتى لو كانت لشكوى طبية مشروعة مثل الألم ؛ أو تناول دواء للشعور بالنشوة " ((NIDA, 2020

يُعرَّف تعاطي الأدوية بشكل خاطىء على أنه استخدام مادة لغرض لا يتوافق مع الإرشادات القانونية أو الطبية (منظمة الصحة العالمية ، 2006)

**إذا كنت تعمل أو تتدرب في صيدلية مجتمعية ، فيرجى إخبارنا إذا كنت ترغب في المشاركة في هذا الاستبيان. إن مشاركتك في استكمال هذا الاستبيان محل تقدير كبير**

توافق على المشاركة

لا أوافق على المشاركة

**الجزء 1. معلومات الديموغرافية للمشاركين**

- **الجنس**
- أنثى
- ذكر
- **العمر (بالسنوات).............................**
- **أعلى مستوى تعلیمي تم الحصول علیه**
- دبلوم
- درجة البكالوریوس
- دراسات علیا
- طالب صیدلة وأتدرب في صیدلیة
- **الحالة الإجتماعیة**
- أعزب/عزباء
- متزوج/ة
- مطلق/ة- أرمل/ة
- **عدد الاطفال**
- لا یوجد اطفال بعد
- 1-3
- 4-6
- >6
- غیر منطبق (أعزب(
- **عدد سنوات الخبرة؟**
- <5
- 5-10
- 11-15
- 16-20
- 20>
- **اي مما یلي یصف تدرجك الھرمي في الصیدلیة**؟
- مالك الصیدلیة
- موظف في صیدلیة
- مالك و موظف في صیدلیة
- متدرب في صیدلیة
- غیر ذلك
- **المحافظة التي تعمل بھا**
- عمّان
- إربد
- الزرقاء
- السلط
- المفرق
- الكرك
- مادبا
- جرش
- عجلون
- العقبة
- معان
- الطفیلة
- **بشكل عام ، ما ھي الطبقة الإجتماعیة الاكثر شیوعاً لعملاء الصیدلیة (بإمكانك اختيار اكثر من إجابة)**
- منخفضة الدخل
- متوسطة الدخل
- مرتفعة الدخل
- **ھل یوجد نادي ریاضي حول الصیدلیة ؟**
- نعم
- لا
- غیر متأكد
- **ھل یوجد أي مركز تجمیل او صالون قریب من الصیدلیة ؟**
- نعم
- لا
- غیر متأكد

**الجزء 2. مدى معرفة و اعتقاد الصیدلاني باستخدام حبوب منع الحمل**

- **وفقًا لمعرفتك ورأیك ، ھل لدى حبوب منع الحمل استخدامات أخرى (غیرتنظیم الحمل)؟**
- نعم
- لا
- غیر متأكد

**إذا كانت إجابتك للسؤال السابق "نعم", أرجو الاجابة على هذا السؤال.**

- **حسب معلوماتك و رأیك , الاستخدامات الاخرى لحبوب منع الحمل قد تشمل:**

|  | صحيح | خطأ |
| --- | --- | --- |
| معالجة حب الشباب |  |  |
| تحفیز نمو الشعر |  |  |
| (غزارة الطمث (نزیف الحیض المفرط |  |  |
| الصداع النصفي المرتبط بالحیض |  |  |
| تشنجات و مغص الحیض |  |  |
| انقطاع الطمث |  |  |
| بطانة الرحم المھاجره |  |  |
| تكسیر البطانة الداخلیة للرحم |  |  |
| علاج غزارة نمو الشعرالغیر مرغوب فیھ |  |  |
| (عسر الطمث (الحیض المؤلم |  |  |
| متلازمة تكیس المبایض |  |  |

- ما ھي الاثار الجانبية لحبوب منع الحمل؟

|  | صحيح | خطأ |
| --- | --- | --- |
| ھبوط الغریزة الجنسیة |  |  |
| تعرق لیلي |  |  |
| زیادة الوزن |  |  |
| تغییرات المزاج |  |  |
| زیادة في حجم الثدي |  |  |
| نمو الشعر غیرالمرغوب بھ |  |  |
| الجلطات الدمویة الوریدیة |  |  |
| سرطان الثدي |  |  |
| سرطان عنق الرحم |  |  |
| أمراض المرارة |  |  |

**الجزء 3أ. خبرة وممارسة الصیدلاني تجاه اساءة استخدام حبوب منع الحمل**

- **وفقا لخبرتك و ممارستك كصیدلاني ،ما ھو الأكثر شیوعًا ؟**

بیع حبوب منع الحمل بوصفة

بیع حبوب منع الحمل بدون وصفة

- **ھل لاحظت أي سوء استخدام / استعمال لحبوب منع الحمل ؟**
- نعم
- لا
- غیر متأكد

**في حال كانت الاجابة نعم للسؤال السابق ارجو الاجابة عن الاسئلة التالية**

- **ما هي تلك الحالات لسوء الاستخدام بين المستخدمين ، ارجو ذكرها**

................................................................................................................................................................................................................................................................................................................

- **ما ھي الفئة الاكثر عرضة لسوء الاستخدام ؟**
- ذكور
- اناث
- ذكور و اناث
- **الفئة العمریة (الأكثر شیوعًا) التي تسيء استخدام حبوب منع الحمل**
- <20
- 20-30
- 31-40
- 41-50
- >50
- **ما هي الفئات الاكثر عرضة لاساءة استخدام ادوية حبوب منع الحمل ؟**
- الغرباء من زائرین الصیدلیة
- المراجعین المعروفین للصیدلیة
- خلیط من الغرباء والمراجعین المعروفین
- **ما هو مصدر المعلومات الذي يوجه مستخدم حبوب منع الحمل لاستخدامها الغير مناسب؟ (يمكنك اختيار أكثر من خيار)**
- صدیق
- السوشیال میدیا و الدعایات
- العائلة
- مركز تجمیل
- مدرب ریاضي
- التلفاز
- صیدلاني
- طبیب
- **حسب خبرتك, أي من المنتجات التالیة عرضة لإساءة الاستخدام ؟(بإمكانك اختيار اكثر من إجابة)**
- Levonorgestrel + Ethinyl Estradiol (e.g. Microgynon)
- Desogestrel+ Ethinyl Estradiol (e.g. Marvelon)
- Norethindrone+ Ethinyl Estradiol (e.g. Kliogest)
- Dydrogesterone+ Ethinyl Estradiol (e.g. Femoston)
- Drospirenone+ Ethinyl Estradiol (e.g. Yasmin)
- Cyproterone+ Ethinyl Estradiol (e.g. Diane)
- Norgestrel+ Ethinyl Estradiol (e,g, Ovral)
- Norethisterone +Estriol + Ethinyl Estradiol (e.g. Trisequens)
- Norethisterone ALONE (e.g. Primolut Nor)

**الجزء 3.ب. موقف الصیدلاني تجاه اساءة استخدام حبوب منع الحمل**

- **في حال تأكد سوء استخدام حبوب منع الحمل, ھل تقوم بصرفھا؟**
- نعم
- لا
- **كیف تتعرف على الذین یسیئون استخدام حبوب منع الحمل ؟ (بإمكانك اختيار اكثر من إجابة)**
- یأتون بانتظام إلى الصیدلیة الخاصة بي (أشخاص معروفین)
- یمكنني التعرف علیھم من انطباعات الوجھ ولغة الجسد
- یطلبون مباشرة ویعترفون باحتیاجاتھم
- لا أستطیع التعرف علیھم
- **إذا لم تكن حبوب منع الحمل موصوفة ، ما الطرق التي من الممكن ان یستخدمھا الصیادلة للحد من اساءة استخدام حبوب الحمل: (بإمكانك اختيار اكثر من إجابة)**
- رفض البیع أو الادعاء أن المنتج غیر متوفر
- تقدیم المشورة و توضیح الاثار الجانبیة لھذه الحبوب
- ابلاغ قسم الیقظة الدوائیة التابع لمؤسسة الغذاء و الدواء
- عمل حملات توعویة و نشرات مخصصة لزیادة الوعي
- طلب وصفة طبیة
- إخفاء المنتج من الرف العادي
- استدعاء الشرطة
- إحالة المریض إلى طبیب
- العمل مع نقابة الصیادلة لحل المشكلة
- عدم القیام بأي شيء
