# Supplementary figures and images for: Unforeseen uses of oral contraceptive pills: Exploratory study in Jordanian community pharmacies
Source: PLoS One. 2020 Dec 21;15(12):e0244373. doi: 10.1371/journal.pone.0244373 (PMC7751968; doi:10.1371/journal.pone.0244373)

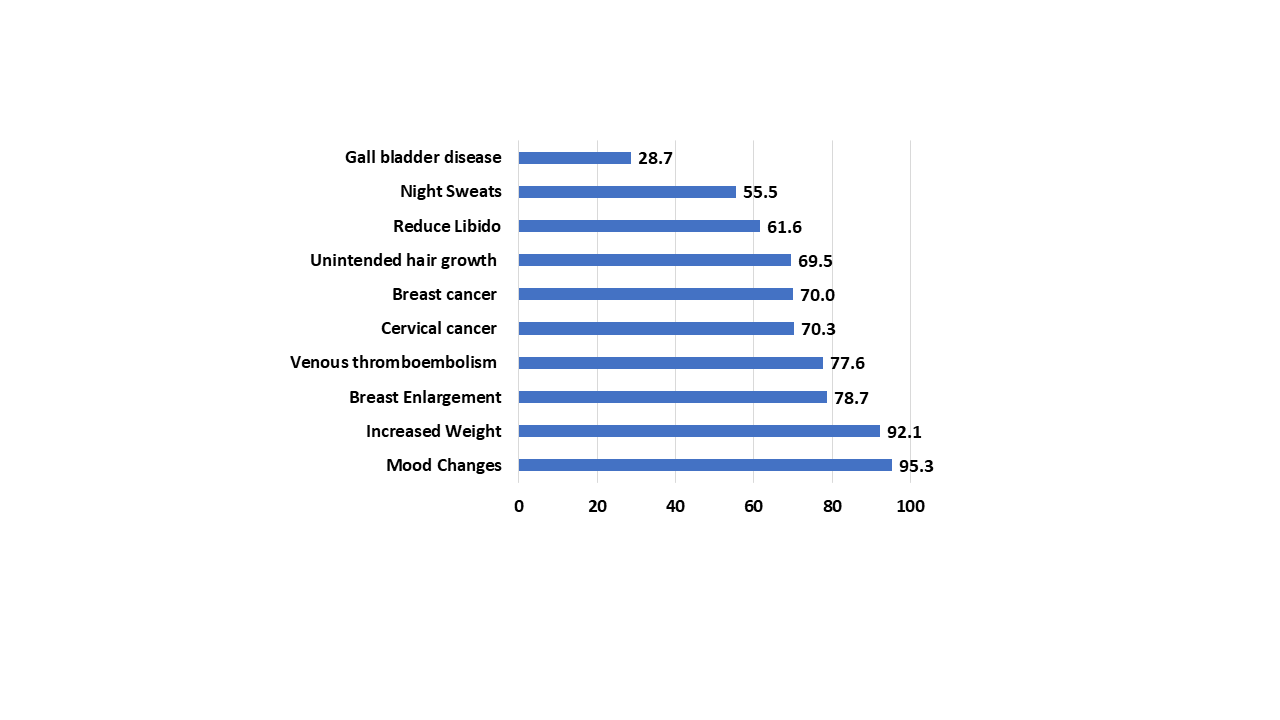

Supplement: S1 Fig — (TIF) [file pone.0244373.s003.tif]
